# The READ-IT study protocol for a feasibility randomised controlled trial of using a support worker/family carer mediated online reading programme to teach early reading skills to adults with intellectual disabilities

**DOI:** 10.1186/s40814-022-00972-x

**Published:** 2022-01-22

**Authors:** Gwenllian Moody, Elinor Coulman, David Gillespie, Mark Goddard, Corinna Grindle, Richard P. Hastings, Carl Hughes, Kate Ingarfield, Zac Taylor, Louise Denne

**Affiliations:** 1 Centre for Trials Research, Cardiff University, Neuadd Meirionnydd, Heath Park, Cardiff, CF14 4YS UK; 2 Centre for Behaviour Solutions, Prospect House, 5 May Lane, Dursley, Glos. Gl11 4jh UK; 3 Centre for Educational Development Appraisal and Research, University of Warwick, Coventry, CV4 7AL UK; 4 Centre for Developmental Psychiatry and Psychology, Monash University, Melbourne, Australia; 5 School of Psychology, Bangor University, Bangor, Gwynedd LL57 2DG UK; 6 Royal Mencap Society, 123 Golden Lane, London, EC1Y 0RT UK

**Keywords:** Intellectual disability, Learning disability, Reading, Adult literacy, Randomised controlled trial, Feasibility study

## Abstract

**Background:**

Many individuals with intellectual disability (ID) have not learnt basic reading skills by the time that they reach adulthood, potentially limiting their access to critical information. READ-IT is an online reading programme developed from the Headsprout® Early Reading (HER®) intervention and supplemented by support strategies tailored for adults with ID. HER® has been successfully used to teach adults with ID to read in a forensic setting by trained staff. The aim of this study is to assess the feasibility of delivering READ-IT to adults with ID by family carers/support workers and will assess whether it would be feasible to conduct a later definitive randomised controlled trial (RCT) of the effectiveness of the programme. The study will aim to contribute to the evidence base on improving outcomes for adults with ID and their caregivers.

**Methods:**

This study is a feasibility RCT, with embedded process evaluation. Forty-eight adults with ID will be recruited and allocated to intervention: control on a 1:1 basis. Intervention families will be offered the READ-IT programme immediately, continuing to receive usual practice and control participants will be offered the opportunity to receive READ-IT at the end of the trial follow-up period and will continue to receive usual practice. Data will be collected at baseline and 6 months post-randomisation.

**Discussion:**

The results of this study will inform a potential future definitive trial, to evaluate the effectiveness of READ-IT to improve reading skills. Such a trial would have significant scientific impact internationally in the intellectual disability field.

**Trial registration:**

ISRCTN11409097

**Supplementary Information:**

The online version contains supplementary material available at 10.1186/s40814-022-00972-x.

## Background

Reading is an essential skill for daily life and a pre-requisite for independent living [1, 2]. Many individuals with ID (known as learning disability in UK health and social care services) have not learnt basic reading skills by the time that they are adults [3] and, as a cohort, have poor literacy skills [2]. An inability to read potentially limits a person’s access to critical information relevant to their daily lives and has been cited as a secondary impact of ID and the cause of significant additional limitations [4].

One of the ways of addressing a lack of reading skills is to make information more accessible using, for example, Easy Read formats. Whilst this may be effective for some, recent research suggests that this is not always the case [5]. Easy Read may be presented in a way that cannot be tailored to meet individual needs [6, 7], and, critically it does not teach a person to read—a skill which may significantly improve that person’s independence, quality of life and overall participation in society [2] with implications both for the person and for those who support them. It should also be noted that what is meant by ‘Easy Read’ is not standardised, including the inclusion or exclusion of text accompanying pictures. Research evidence indicates that young people with ID want the same things as anyone else—to be able to live independently if they so choose, to have friends, a family and to have a job [8]. However, the gap between aspirations and outcomes is much greater amongst people with ID than the wider population and, as they move into adulthood, that gap gets wider [8]. The ability to read is a key to achieving many of these aspirations.

The relationship between levels of proficiency in literacy and employment outcomes for example is well established for the population as a whole [9], and poor literacy skills have been identified as a barrier to employment for people with ID [10]. Even if the ability to read is not a job requirement, it is needed to read job advertisements, complete application forms and to be able to follow procedures and instructions at work.

Making healthy lifestyle decisions is another example. A recently conducted study analysing primary health-care data on 1,424,378 adults found, even when accounting for factors such as neighbourhood deprivation, increased co-morbidity with other healthcare issues and lower mortality rates in the cohort of adults with ID compared to the general population [11]. Reducing this inequality requires initiatives tailored for adults with ID. One of the problems, however, is access to information that might directly empower adults with ID themselves which in turn depends upon health literacy [12]. Many policy initiatives directed at providing that information to the general population are unlikely to benefit people with ID and, as noted above, attempts to increase the accessibility of information are not always effective. This often places a responsibility on carers to mediate access to information.

There is also an emerging body of research that suggests that being able to read can increase the quality of life of individuals with ID helping with additional skills development such as problem-solving, making informed choices and increasing access to the community [2, 13].

There is relatively little research into the reading skills of adults with ID and even less on effective interventions. In part, this has been because of a non-evidence-based perception that it is not possible to teach people with ID to read, whatever their age [14] and more generally that the ability to learn plateaus in adults with ID [15, 16]. Furthermore, the focus of support as children with ID gets older and transition to adulthood often moves away from an academic to more functional curricula with an emphasis on the communication, social and daily living skills deemed necessary for adult life [17]. More recently, these assumptions have been challenged. Studies have shown that it is possible with appropriate teaching and learning strategies, to teach people with ID reading skills [18–20] and, although learning may progress more slowly, that it is possible for adults with ID to continue to learn into adulthood, including learning to read [21, 22].

There has been, however, no high-quality research evidence supported by a randomised controlled trial (RCT) of the effectiveness of strategies to teach adults with ID to read. Much of the research into improved reading skills is with typically developing children. In the UK, the education endowment foundation (EEF) recommends implementing a systematic phonics programme for children, and, because learning to read is not an innate ability, Gough and Hillinger [23] recommended teaching strategies include repeated instruction and opportunities to practice learning to decode text. A systematic review of the literature of teaching strategies to improve reading skills in people with ID concluded that intense practice and instruction is needed and that it should be provided ‘explicitly, systematically, and consistently’ and found no RCTs of reading interventions for adults with ID [24].

HER® is an online reading programme which incorporates sight reading and explicit systematic instruction on the three early reading skills involved in decoding that are part of five critical areas of learning to read: phonemic awareness, phonics and fluency (the other two areas being vocabulary and text comprehension). The ability to decode is an essential component to becoming a proficient reader. HER® [25] involves repeated opportunities to practice decoding and sounding out words, working at the pace of the individual and to suit their needs through 80 online episodes/sessions. HER® has been shown to be effective with typically developing children in large scale implementation studies, including a RCT in the USA [26]. A small UK-based RCT also suggests positive outcomes for HER® versus the usual teaching of reading with children with mild to moderate intellectual disabilities in a mainstream school setting [27]. Our pilot research with small numbers of children in special schools and special resource units has suggested that, with the inclusion of some additional support strategies, HER® can also be effective for children with ID [27–29] especially (but not limited to) those children with the following pre-requisite skills: able to speak clearly, can verbally repeat words modelled to them, are capable of following simple instructions, and have basic computer/touch screen skills (i.e. are able to move and click a mouse appropriately—mouse skills can also be directly taught to increase access). Teaching that is delivered online rather than face-to-face may be easier for people of any age with ID to access, offers a learning experience tailored to their needs and may be more cost effective compared to one-to-one instruction from trained professionals. Critically, it also offers access to more people than can be achieved through one-to-one or even small group instruction.

In the first study to explore the use of HER® to teach basic reading skills to adults with a mild ID, the feasibility of running the intervention in a forensic setting was demonstrated and showed improved decoding skills critical to reading and self-concept scores for participants [3]. No adaptations were needed for the online programme, but trained staff were available to supervise the programme and it was easy to schedule it into the working day. However, working in a secure setting is not the same as typical community and social care settings for people with ID.

A manual incorporating additional support strategies that can be used alongside the standard HER® online programme for anyone helping children with ID to read in home or school settings has been developed by the research team. It has been specifically developed for teachers, teaching assistants and parents mediating their pupil/child’s programme, but will be adapted for support workers and family carers working with adults with ID.

There is a current gap in the availability of suitable reading programmes for adults with ID, in the evidence base around teaching adults with ID new skills and, critically, in the potential impact that teaching adults to read has on their ability to access information relevant to healthy lifestyles, independence, informed choice and ultimately quality of life. READ-IT, and the current research proposal, directly address that gap.

## Methods/design

### Objectives/aim

The aim of this feasibility RCT is to assess the feasibility of delivering a reading intervention to adults with ID by family carers/support workers. The study will aim to contribute to the evidence base on improving outcomes for adults with ID and their caregivers. Importantly, the study will inform a potential, definitive RCT of the effectiveness and cost-effectiveness of the programme. The study primary objective is to examine whether READ-IT can be *delivered* successfully by community support workers/family carers. The study secondary objective is to assess whether it would be feasible to conduct a later definitive RCT of the effectiveness and cost effectiveness of READ-IT.

### Study design

The study is a 2-arm, randomised controlled trial, with 1:1 randomisation using randomly permuted blocks, stratified by setting type (family home vs. other social care setting).

The study will be composed of three stages:

### Stage 1: Intervention refinement and development.

A new intervention (READ-IT) will be developed by further adapting the HER® support manual specifically for use with support workers and family carers of adults and detailing a supervision/mentoring process during the intervention delivery. The intervention will be capable of being delivered in full remotely—a critical factor in study development in a COVID-19 environment. Stage 1 will also include the development of a protocol for obtaining informed consent and data collection remotely and an adaptation for online delivery of all measures used in data collection. These and the procedure for obtaining informed consent will be developed and piloted using public and participant involvement (PPI).

### Stage 2: Feasibility study

The intervention arm participants will participate in an online reading programme (HER®) supplemented by additional support strategies tailored for adults with ID. Support workers and carers will receive a half-day training (delivered remotely) and be given a copy of the support manual. All support workers and family carers will in addition be offered bi-weekly ‘phone-in’ help sessions over the duration of the intervention. The control arm participants will experience usual practice in relation to the support of their reading and will have access to the (HER®) programme after 12 months; however, HER® training or mentoring will not be available to the control arm participants. Baseline measures for all participants will be conducted remotely prior to randomisation and repeated 6 months post randomisation. Selected participants will be approached 6 months post randomisation to take part in a qualitative study designed to address the progression criteria that will not otherwise be clear from other data collected.

### Stage 3: Logic model/full trial protocol

The findings from the feasibility study will be used to review and refine a logic model and, subject to the progression criteria being met, will lead to the development of a protocol for a full trial. This will be achieved through additional PPI input and with the advisory group.

#### Study setting

Individuals will be recruited from family homes, independent living and small group settings (e.g. supported living and residential homes). Settings for people likely to be eligible (those with mild to moderate ID) are most likely to be individual (with their family, or in independent living). This is a single site study.

#### Site selection

This is a single site study and will be carried out at University of Warwick, under the supervision of the Chief Investigator.

#### Participant selection

Individuals will be recruited from family homes, independent living and small group settings (e.g., supported living and residential homes). Families will be directed to the study team by service provider organisations in their local area following a flexible multi-point recruitment method including via targeted service provider organisations, practitioner fora, local and national charitable support organisations, local parent carer fora and self-referral. The strategy is aimed to be flexible and collaborative and information will be gathered regarding the most effective participant identification processes to inform a definitive trial. All potential participants will have been provided with a participant information sheet and will have confirmed interest in participating in the study either directly with the service provider organisation or by returning a completed reply slip to the study team. Potential participants will be contacted by study team researchers to arrange a short screening/recruitment interview, via videoconferencing. Participants are eligible for the study if they meet all of the inclusion criteria and none of the exclusion criteria apply.

#### Eligibility criteria

##### Inclusion criteria

Adults administratively defined as having an ID (i.e. through receipt of/being known to services) who:

1. have the capacity to give informed consent
2. have a level of competence in understanding English suitable to access Headsprout® Early Reading programme. This is assessed via a placement assessment that is provided by HER® to assess where within the intervention the individual is best advised to start and assesses upper-reading ability.
3. can sound out words (although *degree* of articulation will not be a factor). (Sounding out words is a requirement of the HER® component of the intervention).
4. have access to appropriate internet-enabled technology
5. either have basic mouse skills, or the capacity to be taught basic mouse skills
6. are living in a setting in which they are getting daily living skills support supported by a support worker/family carer
7. have access to a supporter who is themselves able to read and willing to support the individual for the duration of the study

##### Exclusion criteria

Adults with ID with visual impairments are severe enough to limit their access to computer-based technology even with adaptations. Adults with ID whose reading skills are too proficient to benefit from the programme, this is assessed by a placement assessment that is provided by HER®.

#### Intervention

The intervention arm participants will participate in an online reading programme (HER®) supplemented by additional support strategies tailored for adults with ID: READ-IT. HER® has been successfully used to teach adults with ID to read. In a pilot study [3], no adaptations were needed to the online programme. However, the intervention was mediated by trained staff who provided additional support when necessary to the participants. HER® has also been successfully used to teach children with ID, again without any adjustments to the programme itself (which is a commercially available product) but using other additional supports and adaptations. These adaptations have been fully described in a manual, developed by our team, for teachers, teaching assistants and parents mediating the reading intervention. A new intervention (READ-IT) will be developed by further developing the adaptations/support manual specifically for support workers and family carers so that those supporting adults with ID are able, in turn to assist with the reading intervention, and detailing a supervision/mentoring process during the intervention delivery. The intervention will be provided remotely in the participant’s home or day care centre. The adaptation of the support manual will be achieved through a PPI model in collaboration with Mencap who is the social care and PPI partner. The research team will also develop a fidelity framework to identify both the fidelity factors included in the HER® programme itself as well as any additional factors associated with adherence to the support manual and engagement with the supervision/mentoring process.

The HER® programme consists of 80 online episodes delivered in sessions of approximately 20‑25 min. HER® recommends between 3 and 6 sessions of 20 to 25 min per week. READ-IT will therefore be delivered on average 16‑20 weeks. Following recruitment and randomisation, support workers/family carers in the intervention group will be invited to attend a half-day remote training workshop. The purpose of training will be to demonstrate how the HER® online programme works and how the support manual can be used by support workers/family carers to help the person that they are supporting. Two options for training dates each month will be offered. Support workers and carers will be given a copy of the support manual and a unique code to access the HER® programme. All support workers and family carers will, in addition, be offered bi-weekly phone-in help sessions over the duration of the intervention. The intervention for each participant will begin once their support worker/family carer has completed the training.

#### Usual practice/comparator

The comparator intervention will be usual practice (UP) with waitlist READ-IT. However, no HER® training or support will be available to the control arm participants during the study period.

#### Retention strategy

To maintain engagement, encourage retention and to thank participants for their time, £20 per participant will be provided per adult during both the initial survey and again at the 6-month point. Support workers/family carers will also be offered £10 during both the initial survey and again at the 6-month point [30]. Participants taking part in qualitative interviews will also be provided with a £20 voucher to thank them for their time [30]. Contact details will be collected during recruitment, and participants will be reminded by email and text message when a data collection follow-up is due.

#### Sample size calculation

A total of 48 individuals will be recruited (randomising 24 per arm). As this is a feasibility study, and the purpose is to provide estimates of key parameters for a future trial rather than to power the current study to detect statistically significant differences, a formal a priori power calculation will not be conducted [31]. However, recruiting 48 participants will provide a certain level of precision around a 95% confidence interval. For example, if 80% of participants provide outcome data at follow-up, the 95% confidence interval around the percentage can be estimated within +/− 11% (i.e. 69 to 91%).

#### Outcomes—spirit figure

The study primary objective is to examine whether READ-IT can be *delivered* successfully by community support workers/family carers. The feasibility of using a range of established outcome measures, proposed to test the intervention in a main trial, will be assessed:

1. Dynamic indicators of basic early literacy skills (DIBELS) which assesses the decoding skills involved in reading
2. A measure of reading self-efficacy (and carer efficacy in supporting the person to read), these will be designed as part of the patient and public involvement (PPI) workshops
3. Quality of life measures for the person with ID: EQ5D-3L (health-related quality of life), the personal well-being index intellectual disability version, completed by the person with ID and the family member/support staff member
4. The version of the client service receipt inventory (CSRI) used in recent ID trials will be used to examine the feasibility of collecting these data for a future health economics analysis, primarily from carers/support staff

The following will also be assessed:

5. Adherence to the READ-IT intervention
6. Fidelity of READ-IT intervention delivery and the most effective measure to assess fidelity

Please see Table 1 for details and timings of all outcome measures (SPIRIT figure) and Additional file 1 for SPIRIT checklist.

- The study secondary objective is to assess whether it would be feasible to conduct a later definitive RCT of the effectiveness and cost effectiveness of READ-IT. The secondary objective will be assessed by reviewing: Recruitment rates and effectiveness of recruitment pathways and randomisation
- Study retention rates
- Assessment of the barriers and facilitating factors for recruitment, engagement and intervention delivery from the perspective of all stakeholders
- Measurement of usual practice
- Acceptability of the primary outcome measures

**Table 1:** Participant timeline (SPIRIT figure): schedule of enrolment, interventions and assessments

Timepoint	Study period			
	Screening	Baseline	Randomisation	Follow-up 6 month post-randomisation
Enrolment				
Eligibility	X			
Informed consent		X		
Contacts data	X			
Randomisation allocation			X	
Assessments				
Demographic data		X		X
Dynamic indicators of basic early literacy skills (DIBELS) completed by study research assistant (S-RA) in response to answers given by participant		X		X
Reading self-efficacy completed by S-RA in response to answers given by participant		X		X
Carer supporting reading self-efficacy		X		X
EQ5D-3L completed by participant		X		X
The personal well-being index intellectual disability completed by S-RA in response to answers given by participant		X		X
Client service receipt inventory (CSRI) completed by family member/support worker		X		X
Qualitative study—participants				X
Qualitative study—support staff/family carers				X

#### Participant flow/ procedure

**Fig. 1:**
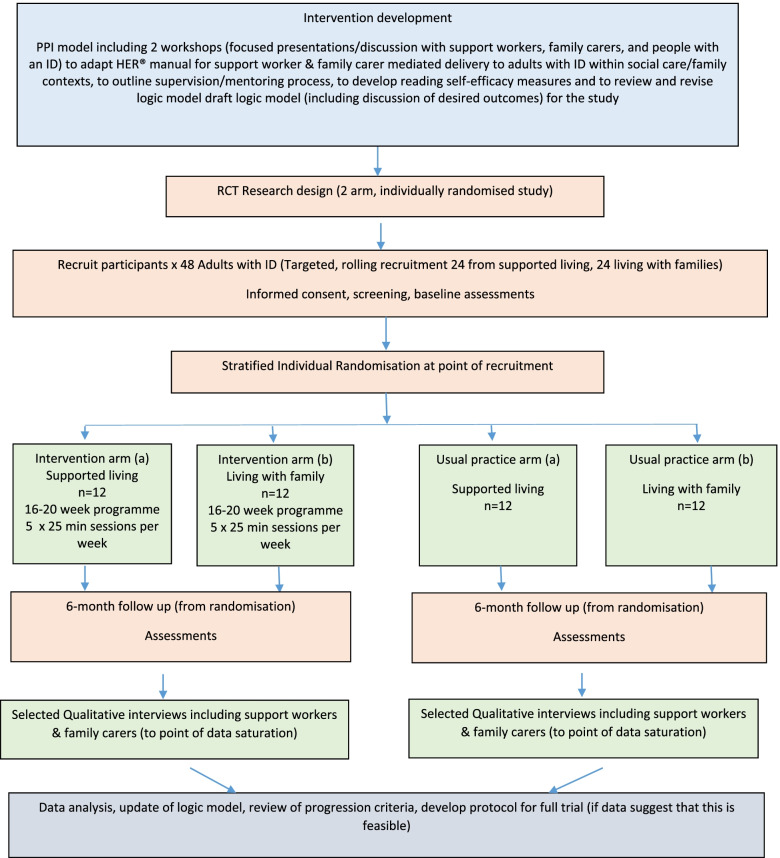
Study flowchart

Figure 1 illustrates the study flowchart.

#### Data collection methods

##### Participant identification

The main strategy for recruitment is to contact those social care provider organisations. Social media advertising will be utilised. Local ID charity organisations and parent carer fora (through the National Parent Carer Forum) will also be contacted. It is expected that settings for people likely to be eligible (those with mild to moderate ID) are most likely to be individual (with their family, or in independent living). Thus, a cluster randomised design is unlikely to be relevant. However, as there is a small risk of contamination in group settings using this design, only one adult with ID and their support worker per group setting will be recruited. During recruitment, a record will be kept of the number of instances in which there is more than one person eligible and interested in taking part within the same setting. This issue will also be explored in the qualitative interviews with support staff working in group settings. These data will inform the choice of research design for a future definitive trial.

##### Screening, recruitment and consent

In order to detect any biases from differential recruitment, a log of all participants considered/approached, including details of the recruitment pathway (via social media or via provider agencies) and whether they are ineligible or eligible will be completed. Provider agencies will be asked to complete a log of the number of potential participants they contact about the study. Both the adult with ID and their family carer/support worker will be consented into the study. There will be two versions of the participant information sheet (PIS), one will be provided to the family carer/support worker and one will be a version utilising images to assist with understanding will be provided to the adult with ID (participant). The participant and family carer/support worker will have been sent the participant information sheet and consent form prior completing any measures and given sufficient time to discuss the information with their support worker/family carer. The study will be explained in detail, including randomisation and consent for long-term follow-up. A placement assessment that is provided by HER® to assess where within the intervention the individual is best advised to start will be used for eligibility screening across all participants prior to baseline data collection. Consent will be gained for this eligibility assessment. If a participant is happy to take part, informed consent will be obtained. Consent will be taken either face-to-face or via videoconferencing. The study research assistant (S-RA) will read aloud each statement of the consent form and ask the participant to agree to each statement and approve that each one is signed individually. The S-RA will then sign on the participants’ behalf if this process is completed virtually. Once consent is gained, the following will be completed:

- A contacts form will be completed for participants including multiple methods of contact (address, telephone, email address) to minimise loss to follow-up.
- Baseline data collection completed (either at time of recruitment or at a suitable time for the participant). This will either be completed face-to-face or virtually via teleconferencing.

The addition of the option of completing consent and data collection virtually was included as a result of COVID-19 restrictions.

#### Randomisation

Participants will be randomised following screening and completion of baseline assessments. Participants will be randomised in a 1:1 ratio using a block randomisation programme developed by the Centre for Trials Research (CTR). Allocations will be balanced by setting type (family home vs. other social care setting). Participants will be randomised to READ-IT in addition to usual practice or Usual Practice alone (i.e. for their reading from those within their care environment). The research assistant providing on-going intervention support (this must not be the S-RA collecting baseline and follow-up data as they should remain blind to allocation, the intervention research assistant (I-RA)) will inform participants and their support workers/family carers of their allocation by telephone and will provide all details of starting the READ-IT programme to those allocated to the intervention arm. Randomisation will be performed by the study manager/data manager who will inform the I-RA of the allocation prior to their telephone call with the participant. Given that no more than one individual from a group setting will be recruited to the study, there is no danger of participants from the same setting being randomised into different trial arms, limiting the risk of contamination.

#### Frequency and duration of follow-up

Data will be collected at 6 months post-randomisation. Participants will be contacted by the S-RA to complete this face-to-face or via teleconferencing. To reduce the risk of bias, the S-RA will read questions from the questionnaire directly, remain blind to the participants’ allocation and will ask participants not to reveal their allocation. If allocation is revealed, this will be noted.

#### Process evaluation

A process evaluation will be based on the MRC framework [32] and will incorporate data from the interviews, recruitment pathways and fidelity/adherence data to examine five key aspects of the feasibility of conducting a definitive trial of HER® for adults with ID: (1) intervention recruitment, adherence and reach; (2) intervention implementation; (3) intervention mechanisms, including receipt and acceptability; (4) the impact of COVID-19 on service as usual and (5) the feasibility of implementing HER® within a definitive RCT.

#### Data management and security

Study data will be entered on to paper case report forms (CRFs) by the S-RA at the time of data collection and subsequently entered on to a MS Access Database directly by the S-RA. A sample of CRFs will be scanned and checked visually on receipt by the study administrator, data manager or study manager. RAs will be trained in good clinical practice (GCP) and study specific processes. Hard copies of personally identifiable and research data will be held separately and securely in a locked cupboard, with access limited to essential research team members. CRFs will be pseudonymised and data entered manually onto a secure, password-protected Microsoft SQL database by the study administrator (SA) and data queries noted. Ten percent of all data will be quality checked and all data queries actioned by the data manager (DM). Any key data queries will be taken to the study management group (SMG) or SSC as appropriate. Wherever possible, data will be validated at point of entry, thereby reducing the opportunity for missing or unexpected data. All changes made to the data will be recorded and visible via an audit log within the database. Finally, data will be checked during data cleaning using SPSS syntax for validations and missing data. Qualitative interviews will be conducted remotely, recorded via the encrypted services offered by the platform used and stored on password-protected computers at site. Recordings will be securely transferred to the study team at the CTR by Fastfile or courier. All files will be encrypted and transcripts will be fully pseudonymised prior to analysis. Data security and confidentiality will be ensured, in line with GDPR. A data management plan will be completed and adhered to. Only the trial team will have access to the final study dataset.

#### Statistical methods/analysis plan

The majority of outcome analysis will be descriptive in nature. Continuous data will be reported as means and standard deviations, or medians and interquartile ranges, as appropriate. Categorical data will be reported as frequencies and proportions. All data will be reported both overall, per arm and by setting type. Outcomes will be estimated with their associated 95% confidence intervals. No formal hypothesis testing will take place. A detailed statistical analysis plan will be written and agreed by the study management team prior to any analysis taking place. The estimates obtained from the feasibility questions will be used to inform the design, sample size, randomisation strategy and analytical approach for a definitive effectiveness study. The findings from the study will be reported in line with the CONSORT extension for pilot and feasibility studies [33].

#### Cost effectiveness methods/analysis plan

Whilst no formal economic analysis will take place, consideration will be given to the practicalities and difficulties associated with collection of quality of life and CSRI data that would be needed in a future trial.

#### Qualitative methods/analysis plan

Semi-structured qualitative interviews will be conducted with a selection of adults with ID, support workers and family carers delivering READ-IT, after the 6-month follow-up assessment. Sufficient interviews will be conducted to achieve ‘information power’ [34] which focuses on the quantity and quality of information gathered relevant to the research question rather than sample size, but is likely to include 8 to 12 adults with ID with similar numbers of support workers and family carers. Thematic analysis as outlined by Braun and Clarke [35] will be used to analyse the data, with a focus on identifying patterns of shared meaning.

#### Progression criteria for a definitive trial

Criteria will inform the decision to progress to a definitive trial, with consideration to issues that may have affected meeting any of these criteria and steps that can be taken to overcome these issues within a full trial. These will be based on a traffic light system with green indicating ‘go without any modification necessary’; amber indicating ‘potential proceed to definitive trial, remedying early issues’; red indicating ‘stop’.

- **Participant recruitment**: % of participants approached, and who are eligible, consent to the study (and thus are willing to be randomised)
  Green ≥50%
  Amber 30≥<50%
  Red <30%
- **Individual randomisation possible** (% of total number of settings in which more than one participant is eligible and willing to take part) (NB. Amber/red here may lead to a proposal for a cluster randomised design)
  Green ≤20%
  Amber 20>≤40%
  Red >40%
- **Rate of recruitment**: % of recruitment target (48 participants) are recruited within the study recruitment period
  Green 100%
  Amber 70≥<100%
  Red <70%
- **Participant retention**: % of participants retained 6 month follow-up data collection timepoint
  Green 75<>100%
  Amber 50≥<70%
  Red <50%
- **Usual practice**: % of participants in the UP arm of the study who receive an alternative structured programme designed to teach them to read between baseline and 6 month follow-up
  Green ≤30%
  Amber 30>≤50%
  Red >50%
- **Fidelity**: Self-rating forms indicate % of READ-IT manual components have been met both across and within sessions.
  Green 70<>100%
  Amber 50≥<70%
  Red <50%
- **Adherence**: % of participants and their support workers/family carers who adhere to the READ-IT programme (attend training, complete 80 episodes within 20 weeks, meet adherence criteria built into HER® programme)
  Green >70%
  Amber 50≥<70%
  Red <50%
- **SSC consensus**—considering all progression criteria, feasibility study findings and evidence of whether progression criteria not met can be mitigated, a clear majority of the SSC independent members recommend progression to a definitive trial

#### Adverse event reporting

There are no expected adverse events related to the intervention or research procedures; the NHS Health Research Authority, London - Camberwell St Giles Research Ethics Committee have approved that adverse events should not be reported for this study.

#### Auditing

No independent audits are planned.

#### Study governance

Ethical approval for this study was given by the NHS Health Research Authority, London - Camberwell St Giles Research Ethics Committee on 3 December 2019, reference number 19/LO/1784. Any protocol amendments will be approved by the NHS Health Research Authority, London - Camberwell St Giles Research Ethics Committee. A SSC will meet approximately two to three times over the course of the study to provide oversight. The SSC will consist of an independent chair with expertise in ID research and trials research, an independent ID expert/clinician, independent statistician and a family carer representative (family member of adult with ID).

#### Confidentiality

All data will be kept for 15 years in line with Cardiff University’s Research Governance Framework Regulations for clinical research. Electronic data will be stored confidentially on password-protected servers maintained on University networks. All hard copy forms will be stored in locked filing cabinets. For participant interviews, all audio files will be recorded on encrypted audio-recorders and securely held in password-protected servers maintained on University networks. Audio files will be transcribed and pseudonymised using University-approved transcription companies. No identifiable data will be published.

#### Dissemination policy

A publication plan and dissemination policy will be written. Outputs from the READ-IT feasibility study will include open access peer reviewed journal articles in international academic journals, at national and international academic conferences at University public engagement events and a lay summary of the results will be included on the CTR and University of Warwick websites. The results of the study will also be disseminated to all participants. The READ-IT team will work in partnership with Mencap for dissemination to stakeholders including commissioners and policy makers. Dissemination events will be arranged for key stakeholders and policy makers. Any data requests should be made to the CTR. The CTR is a signatory of AllTrials and aims to make its research data available wherever possible.

#### Public involvement

The adaptation of the support manual will be achieved through a PPI model in collaboration with Mencap who is the social care and PPI partner. This will involve two workshops with adults with ID and their support workers/family carers. The PPI workshops will be used to refine a logic model for the intervention and to develop a measure of reading self-efficacy for adults with ID which is grounded in everyday life. A mirror version of this measure will be provided for support workers/family carers. An advisory group with members recruited from the PPI workshops will be established to review the findings of the study, progression criteria and key issues in the protocol for a full trial. The SSC will include an independent lay representative who is a family member of an adult with ID.

## Discussion

The current health/social care context suggests that research into skills development in adults with ID is timely. For example, the recently published NICE guidance learning disabilities and behaviour that challenges: service design and delivery [36] reflects current policy in the support of people with ID in England with a focus on providing support services in the community. It continues to build upon the model of care outlined in the Mansell Report [18] as well as the transformation programme set out in Transforming care: A national response to ‘Winterbourne View Hospital’ [19]. The policy programme’s goal is to drive system-wide change and enable more people to live in the community, with the right support, and close to home with a specific aim to reduce the number of beds for people with a learning disability in mental health hospitals 35% to 50% by 2019. This requires not only a focus on developing enabling communities [20] but also on supporting individuals with ID to live in their communities, access services and teaching them the necessary skills to be active participants within these. The READ-IT logic model directly addresses this need by targeting reading—a critical skill. The results of this study will contribute to the evidence base on teaching adults with ID to read and will be used to inform a potential future definitive trial, to evaluate the effectiveness of READ-IT to improve reading skills. Such a trial would have significant scientific impact internationally in the intellectual disability field.

## Supplementary Information

**Additional file 1.**

## Data Availability

Not applicable—protocol paper.

## Abbreviations

CRFs: Case report forms
CTR: Centre for trials research
CSRI: Client service receipt inventory
DM: Data manager
DIBELS: Dynamic indicators of basic early literacy skills
EEF: Education endowment Foundation
GCP: Good clinical practice
HER®: Headsprout® Early Reading
ID: Intellectual disability
I-RA: Intervention research assistant
PPI: Public and participant involvement
RCT: Randomised controlled trial
SA: Study administrator
SMG: Study management group
S-RA: Study research assistant
SSC: Study steering committee
UP: Usual practice
